# Comparison of Mendeliome exome capture kits for use in clinical diagnostics

**DOI:** 10.1038/s41598-020-60215-y

**Published:** 2020-02-24

**Authors:** Reuben J. Pengelly, Daniel Ward, David Hunt, Christopher Mattocks, Sarah Ennis

**Affiliations:** 1 Human Genetics and Genomic Medicine, Faculty of Medicine, University of Southampton, Southampton, UK; 2 National Genetics Reference Laboratory (Wessex), Salisbury District Hospital, Salisbury, UK; 3 Wessex Clinical Genetics Service, Princess Anne Hospital, Southampton, UK

**Keywords:** Genetics, Genetics research

## Abstract

Next generation sequencing has disrupted genetic testing, allowing far more scope in the tests applied. The appropriate sections of the genome to be tested can now be readily selected, from single mutations to whole-genome sequencing. One product offering within this spectrum are focused exomes, targeting ~5,000 genes know to be implicated in human disease. These are designed to offer a flexible platform offering high diagnostic yield with a reduction in sequencing requirement compared to whole exome sequencing. Here, we have undertaken sequencing of control DNA samples and compare two kits, the Illumina TruSight One and the Agilent SureSelect Focused Exome. Characteristics of the kits are comprehensively evaluated. Despite the larger design region of the Agilent kit, we find that the Illumina kit performs better in terms of gene coverage, as well as coverage of clinically relevant loci. We provide exhaustive coverage statistics for each kit to aid the assessment of their suitability and provide read data for control DNA samples to allow for bioinformatic benchmarking by users developing pipelines for these data.

## Introduction

Next-generation sequencing (NGS) has proven to be a disruptive technology in genetic research and diagnostics, allowing the sequencing of genomic regions orders of magnitude larger than that routinely possible using traditional technologies such as Sanger sequencing, leading to increased diagnostic rates^1–3^. Pre-sequencing target capture, enriching a defined genomic region of interest, allows for the particularly cost-effective application of NGS to patient samples, with a range of capture platforms available for this^4^.

With the range of NGS based technologies available, selecting the appropriate tool requires a balance of the scope of data acquisition with analytical requirements. Where data on more regions of the genome is captured, this increases the likelihood of capturing the aetiological variant(s) in the patient however sequencing, data analysis and interpretation costs are all increased by the larger dataset. Conversely, small targeted panels are cost efficient and comparatively trivial to analyses, though there is an increased chance of the aetiological variant(s) not being captured^5^. The use of capture kits introduces biases and limitations into the data, and therefore the data even for the target genes is sometimes sub-optimal when compared to whole-genome sequencing data^6^.

Whilst NGS based testing allows the inclusion of more genes, often the quality of data and coverage for a given gene is poorer than for a single gene Sanger sequencing-based test, so care must be taken in the application of NGS based tests in certain scenarios. As with the use of any clinical testing methodology, it is essential that appropriate quality assurance procedures are in place prior to the utilisation of a method for diagnostics^7^. This includes the validation of a tool, and verification that the tool is performing as expected^8,9^.

Many studies have been performed to quantify the diagnostic yields possible through the utilisation of whole genome and whole exome sequencing (WGS and WES respectively). A recent meta-analysis showed the diagnostic utility to be 42% for WGS and 38% or WES in studies published in 2017^2^. This mere 4% difference in diagnostic rate highlights the increased density of pathogenic variants in the exome, whilst also reflecting our ability to better interpret coding variants and assign pathogenicity to them^10^.

Capture platforms for focused exome sequencing (FES) have been introduced, which target the ~5,000 genes that have been implicated in human disease, often termed the ‘Mendeliome’. This has the specific advantage of requiring the generation of less sequence data in order to obtain sufficient depth of coverage across the region of most clinical interest for diagnostic purposes compared to whole genome sequencing. Commercial platforms for this include the TruSight One kit (TSO; Illumina, San Diego, CA, USA) and the SureSelect Focused Exome kit (SSFE; Agilent, Santa Clara, CA, USA).

Here we compare data generated with two commercially available FES kits, the Illumina TSO and Agilent SSFE, and have assessed their utility and suitability for implementation in routine clinical genetic testing.

## Results

### Read sequence biases

We assessed the biases in base composition along the reads, as biases in this composition can be introduced during DNA fragmentation^11–14^. Both the SSFE and TSO kits do exhibit a bias, however TSO has a greater degree of deviation from the expected frequency in the first 20 bases of the read (p < 0.001, mean residual from 25% of 5.0% and 7.2% for SSFE and TSO respectively (Supplementary Figure 1); this is as expected owing to the enzymatic fragmentation for TSO.

### Target regions concordance and coverage

Data output and alignment statistics averaged across the 12 samples sequenced for each kit are shown in Table 1. A higher mean coverage across the target region is seen for the TSO samples, owing largely to the smaller target region. The SSFE data shows greater specificity of capture, having fewer off target reads (28% for SSFE compared to 35% for TSO for reads mapped outside of the target region ± 150 bp).

**Table 1:** Summary coverage statistics for triplicate sequenced samples.

		SSFE		TSO	
		Mean	CV^a^ (%)	Mean	CV (%)
Read generated		86,509,698	8.1	80,712,320	17.7
Reads mapped		84,816,034	8.0	74,590,089	18.0
Duplicate reads		21,918,522	14.0	19,674,723	24.0
Reads mapped to target		46,466,309	5.9	38,474,359	15.6
Reads mapped to target ± 150 bp		61,283,075	6.0	48,581,312	15.9
Mean coverage of target		211	5.8	317	15.2
Target^b^ size (bp)		17,846,036	—	11,946,514	—
Target^b^ covered (%) with read depth≥:	1 X	100	0.1	99	
	5 X	100	0.1	99	0.1
	10 X	99	0.1	98	0.2
	20 X	98	0.3	98	0.3
	30 X	97	0.5	97	0.4
	50 X	92	1.1	96	0.8
	100 X	76	2.8	91	3.0

^a^Coefficient of variation.

^b^Target regions as defined by vendor target BED file.

**Figure 1:**
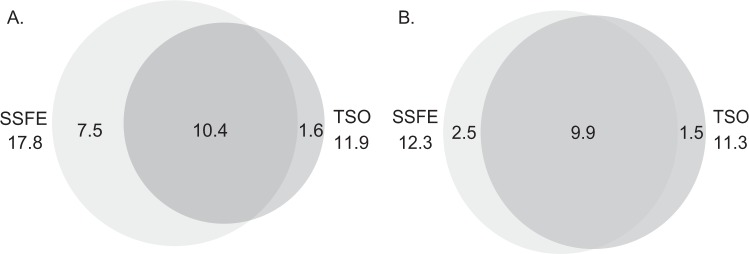
Euler diagram showing size (Mb) of regions as defined by the vendor provided target locations (**A**) and RefSeq transcripts covered to ≥ 20 X in our data without regard for vendor defined targets (**B**).

**Figure 2:**
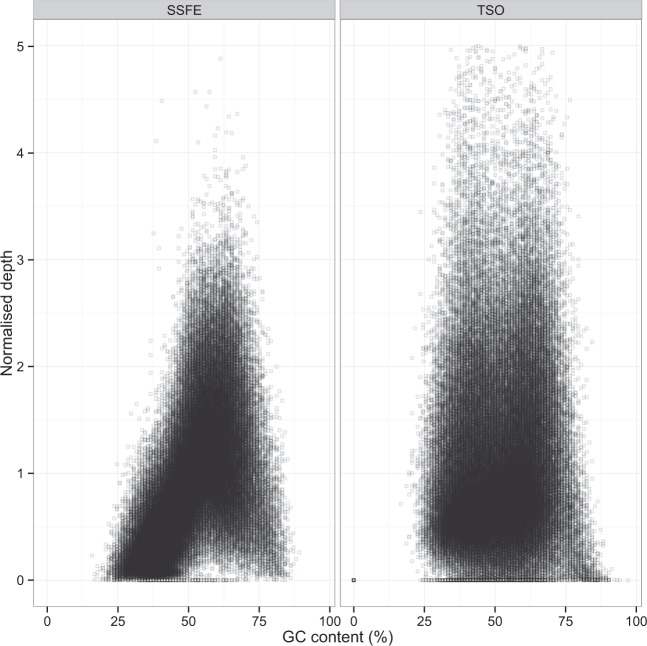
Capture biases for the two kits. The degree of GC bias seen is greater for the SSFE data (r^2^ = 0.56 *vs*. r^2^ = 0.09 for SSFE and TSO respectively, p < 2.2×10^−16^ for both, Pearson’s correlation).

The SSFE kit has 17.8 Mb of targeted regions in total, 50% larger than the TSO capture target; in terms of transcript coverage however the SSFE kit covers just a 9% larger coding region covered to ≥ 20 X(Fig. 1). There is ~10 Mb of the genome included in the target design of both, and a similar amount of the transcribed genome covered to ≥ 20 X between the two kits. Both kits demonstrate high coverage consistency between samples (r > 0.99 for base-wise coverage for both). SSFE exhibits a greater degree of GC bias (Fig. 2), with a stronger positive correlation between GC content and read depth than TSO.

### Specific region of interest coverage

The most useful measure of coverage for evaluation of a kit for genetic testing is the coverage of genes. Both manufacturers provide lists detaining the genes considered in the design of the panels, with SSFE having 20% more genes listed (Table 2; Supplementary data). Despite this, the TSO kit covered more genes to a median depth of 20 X across 0.99 of the genic regions, and a greater number of HGMD variants.

**Table 2:** Region of interest coverage.

		SSFE	TSO
Genes listed		5,576	4,663
Genes with proportion covered	> 0.99	3,774	3,970
	0.95 < x ≤ 0.99	597	368
	0.90 < x ≤ 0.95	349	151
	0.80 < x ≤ 0.90	237	117
HGMD^a^	Disease causing (133,378 total)	125,000	128,784

^a^Human Gene Mutation Database release 2015^23^

**Figure 3:**
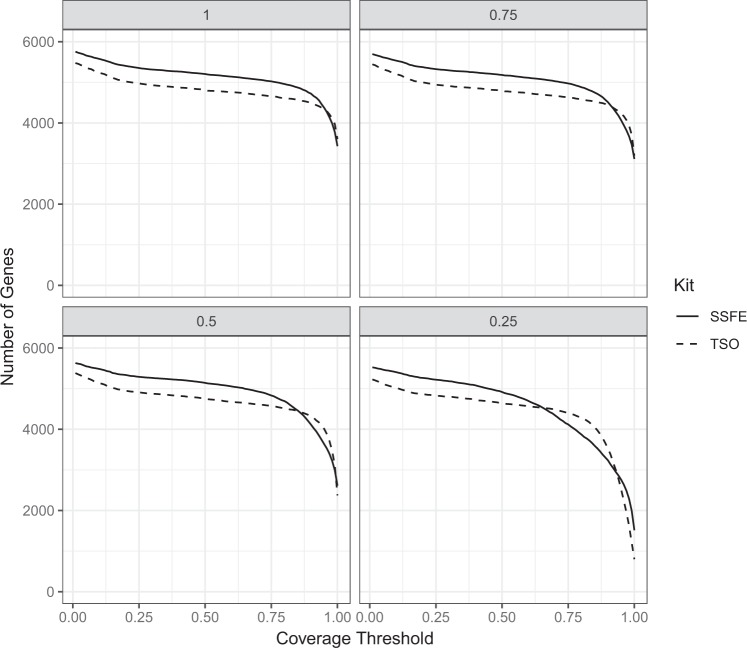
Proportion of genes covered to varying levels in downsampled datasets representing 12–48 samples being included in a single sequencing run. A reduction in the number of genes covered to a high level can be seen with the data down-sampling.

We performed down-sampling of the data in order to determine the expected coverage where greater numbers of samples were included in a batch, as would be the case for a sustainable sequencing service. For both kits, there is a reduction in the number of genes covered (Fig. 3). TSO demonstrates increased robustness of gene coverage with down-sampling.

### Variant detection sensitivity

The variant detection sensitivity and precision were quantified (Table 3). The SNP sensitivity and precision were > 0.99 for both kits, with no significant difference in the sensitivity. For indels, SSFE performed better than TSO in terms of accuracy. SSFE identifies ~1.9-fold more SNPs than TSO, owing to the larger region of the genome captured; however, it is of note that TSO covered to 20 X a marginally greater number of variants contained in the HGMD dataset, capturing the clinically important regions.

**Table 3:** Sensitivity and precision of variant detection for the two capture kits.

		SSFE		TSO	
		Mean	CV	Mean	CV
SNP	Count	10,883	—	5,798	—
	Sensitivity	0.995	0.08%	0.996	0.04%
	Precision	0.994	0.05%	0.997	0.01%
Indel	Count	626	—	178	—
	Sensitivity	0.791	0.71%	0.757	0.43%
	Precision	0.869	0.48%	0.766	2.72%

## Discussion

We have performed a comprehensive technical evaluation of two focused exome sequencing capture kits. The choice of kit for a particular purpose will depend upon the exact purpose intended, particularly requirements for the gene content. We have provided exhaustive coverage statistics for each kit to aid the assessment of suitability and provide read data for control DNA samples to allow for bioinformatic benchmarking by users developing pipelines for these data.

Aside from kit performance, it is worth also considering practicalities of using these kits in practice such as reagent cost and hands-on time for sample processing. List prices for the two kits are similar per sample, equating to £135 GBP and £142 per sample (TSO and SSFE respectively) for sample processing up to library preparation, and £109 for sequencing (as of January 2020, assuming 36 samples per run on the NextSeq platform). Both kits also require common molecular laboratory equipment and consumables.

**Figure 4:**
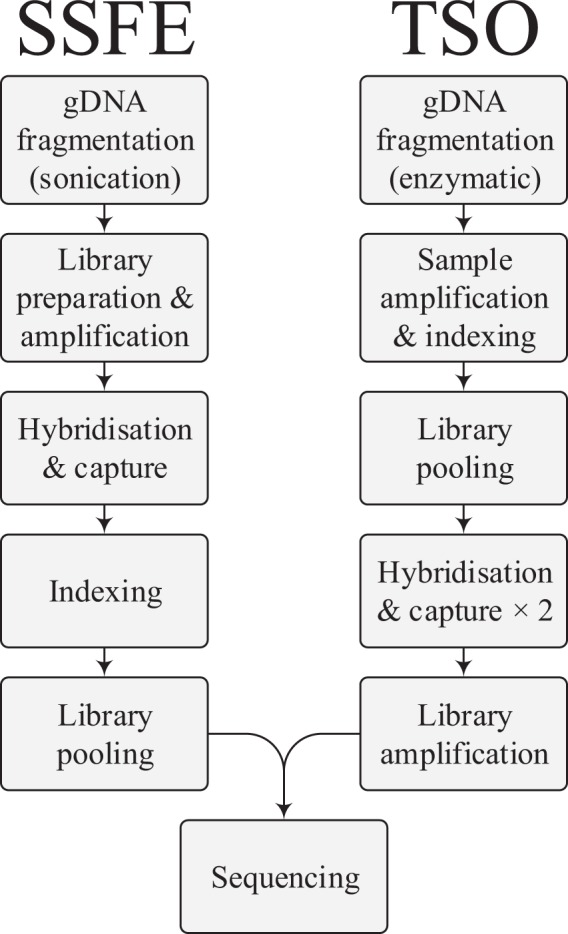
Overview of laboratory processes for the two capture kits. For SSFE physical fragmentation is followed by DNA fragment repair and ligation of adapter sequences; hybridisation of patient DNA with the baits and pulldown is then performed, followed by library indexing and pooling^19^. For TSO, combined fragmentation and adapter ligation is performed enzymatically, followed by sample amplification, indexing and pooling. Two iterations of bait hybridisation and pulldown are then performed, with a final pooled library amplification^18^.

The two kits have dissimilar workflows (Fig. 4), which results in some key practical differences. SSFE requires ~24 hours of lab time, including one overnight hybridisation, whilst TSO requires ~36 hours, with two extended hybridisations, at least one of which is to be overnight. One key difference in workflows is the earlier addition of sequencing indices to the samples in the TSO protocol, with benefits for sample security if any sample switches or cross-contamination occur later in the process.

The use of FES and other NGS platforms in clinical diagnostics affords many real-world advantages over traditional, often single gene, methods. Alongside the high diagnostic yield, there is likely to be a shortened ‘diagnostic odyssey’ for many patients, due to the parallel testing of multiple genes as opposed to in sequence. NGS gene panel which target a small number of genes (~5–100) for a specific disorder are often utilised, requiring the design of a capture kit bespoke to the intended application. This is a non-trivial process, and requires subsequent validation of the laboratory process with any modifications of the bespoke capture kit^15^.

An alternative to this process is the use of FES or similar where the genes are sequenced, but analysis is restricted by the use of ‘virtual panels’, which are more readily modified, without the need for laboratory validation. The ease of gene inclusion in these virtual panels will also allow greater equity of access to genetic testing to those with rare disorders for which it is not viable to establish a bespoke validated test within a clinical testing service.

FES has advantages over WGS due to its amenability to sequencing using lower capacity sequencing platforms. These platforms have cheaper purchase and running costs and are thus more accessible to smaller labs. This scale of testing may therefore be a ‘sweet spot’ for many applications.

## Materials and Methods

Four DNA samples were analysed, being cell line derived DNA from a trio of samples from CEPH/Utah pedigree 1463 (samples NA12878, NA12891 & NA12892; Corriel Institute, Camden; NJ, USA) and NIST reference standard RM 8398 (derived from NA12878; National Institute of Standards and Technology, Gaithersburg, MD, USA)^16,17^. Library preparation was performed using the TruSight One sequencing panel (Illumina, San Diego, CA, USA; part number FC-141-1007) and the SureSelect^XT^ Focused Exome (Agilent, Santa Clara, CA, USA; part number 5190–7788), according to manufacturer instructions^18,19^. Each sequence run included 12 samples, being the 4 analysed DNA sources following library preparation in triplicate.

Library sequencing was performed using a NextSeq 500 (Illumina, San Diego, CA, USA) high output run using v2 chemistry, with a separate run for each library preparation. Paired-end reads of lengths of 150 and 100 bp were generated for the TSO and SSFE libraries respectively in accordance with the supplied protocols. Data were converted to FastQ format using vendor software bcl2fastq v2.16.0.10. Reads were aligned to GRCh37 (hg19) using NovoAlign MPI v2.08.02 (Novocraft, Selangor, Malaysia). Alignments were sorted and duplicates marked using picard v1.97 (https://broadinstitute.github.io/picard/), variant calling was performed using SAMtools v0.1.19^20^ and coverage statistics were collated using BEDTools v2.17.0^21^. A position was considered ‘covered’ if the median depth of coverage across the 12 samples at that base position was ≥ 20 X.

For determining gene coverage, transcripts were defined using the RefSeq database, with the coding region (± 20 bp) of the longest transcript for each gene being considered. Down-sampling the read data was performed by randomly selecting a proportion of the reads prior to alignment. Base composition biases of reads were evaluated using FastQC 0.11.3. (http://www.bioinformatics.babraham.ac.uk/projects/fastqc). Statistical analyses on the base composition data comprised an unpaired t-test on the absolute deviation from 25% for each of the biases. Accuracy of genotype calling was performed on the RM 8398 replicates, comparing against high confidence calls of Genome in a Bottle data (v2.19) for this sample using RTG Tools v3.5.2^22^.

## Supplementary information

Supplementary figure.

Supplementary Dataset 1.

## Data Availability

Raw sequence data have been deposited in the ArrayExpress database at EMBL-EBI (www.ebi.ac.uk/arrayexpress) under accession number E-MTAB-8349.
