# Supplementary material for: Comparison of Mendeliome exome capture kits for use in clinical diagnostics
Source: Sci Rep. 2020 Feb 24;10:3235. doi: 10.1038/s41598-020-60215-y (PMC7039898; doi:10.1038/s41598-020-60215-y)
Supplement: Supplementary file 1 — Supplementary figure. [file 41598_2020_60215_MOESM1_ESM.pdf]

Title: Comparison of Mendeliome exome capture kits for use in clinical diagnostics

Authors: Reuben J. Pengelly[1]*, Daniel Ward[2], David Hunt[3], Christopher Mattocks[2], Sarah Ennis[1]

1. Human Genetics and Genomic Medicine, Faculty of Medicine, University of Southampton, Southampton, UK
2. National Genetics Reference Laboratory (Wessex), Salisbury District Hospital, Salisbury, UK.
3. Wessex Clinical Genetics Service, Princess Anne Hospital, Southampton, UK

*Corresponding. R.J.Pengelly@soton.ac.uk

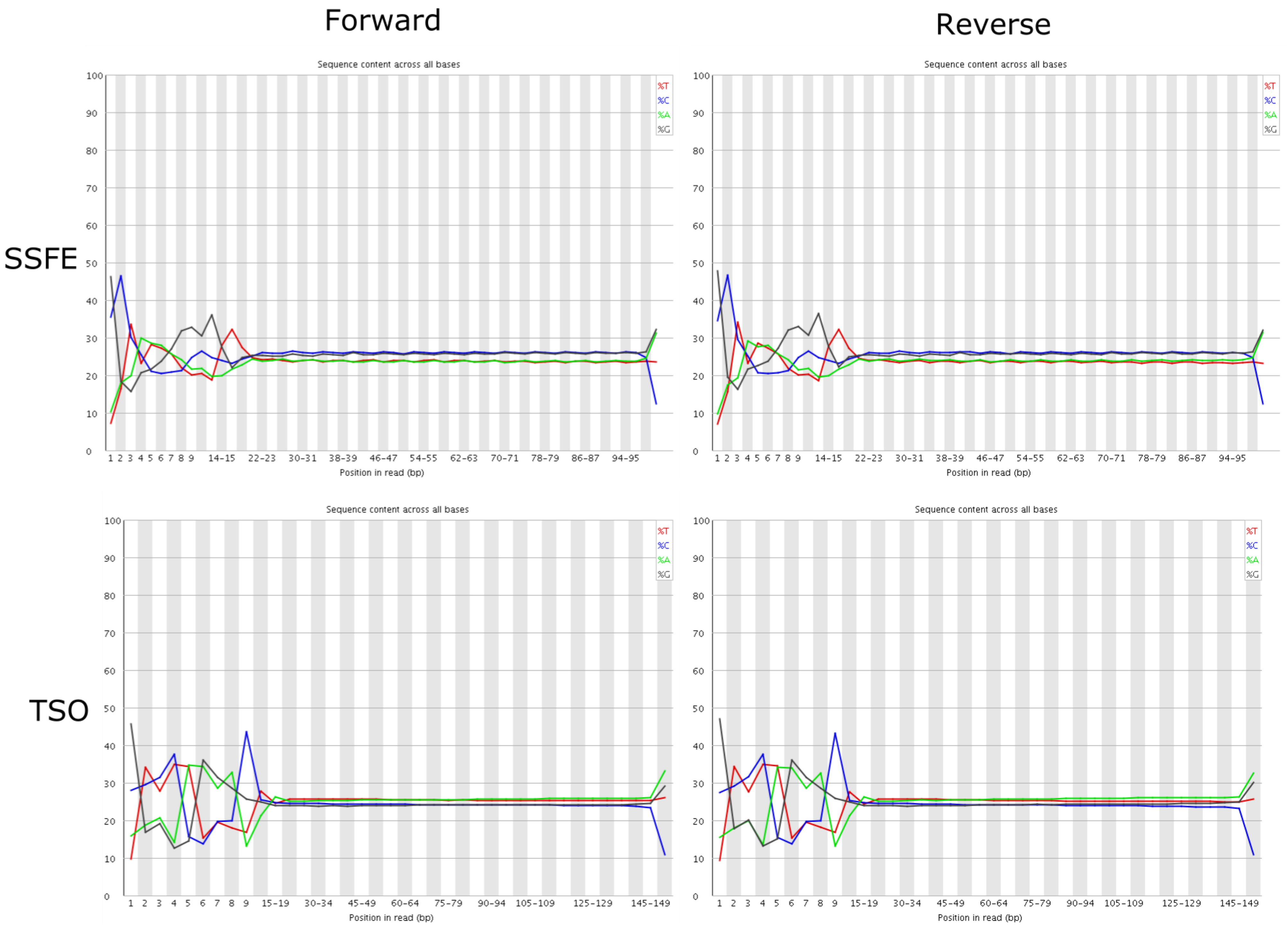
Forward
Reverse
SSFE
TSO
Sequence content across all bases
%T
%C
%A
%G
Position in read (bp)
